# A Clinical and Histological Study about the Socket Preservation in a Patient under Oral Bisphosphonates Treatment: A Case Report

**DOI:** 10.3390/biology10040262

**Published:** 2021-03-25

**Authors:** Antonello Falco, Francesco Bataccia, Lorenzo Vittorini Orgeas, Federico Perfetti, Mariangela Basile, Roberta Di Pietro

**Affiliations:** 1 Falmed Medical Care, Viale G. d’Annunzio, 73, 65127 Pescara, Italy; antonellofalco@yahoo.it (A.F.); lorenzo.vittoriniorg@gmail.com (L.V.O.); 2 Department of Medical, Oral and Biotechnological Sciences, G. d’Annunzio University of Chieti-Pescara, Via dei Vestini 31, 66100 Chieti, Italy; francescobataccia@gmail.com (F.B.); federico186@gmail.com (F.P.); 3 Department of Medicine and Ageing Sciences, G. d’Annunzio University of Chieti-Pescara, Via dei Vestini 31, 66100 Chieti, Italy; r.dipietro@unich.it

**Keywords:** oral bisphosphonates, osteonecrosis of the jaw, extraction socket, socket preservation, bone graft, histological analysis, bone

## Abstract

The aim of the present study is to assess the clinical and histological healing of a post-extractive alveolus following the procedure for socket preservation, in a patient receiving oral bisphosphonates for more than 6 years. After the extraction, enzymatically-deantigenated horse bone granules and an equine pericardium membrane were used to preserve the tooth socket. The patient was placed on a monthly follow-up in order to monitor the healing process. A 3 mm trephine bur was used to drill the bone for implant site preparation and to collect the bone sample. No signs and symptoms related to osteonecrosis of the jaws were reported. Histological data showed that, after 5 months, the mean percentages of trabecular bone, bone marrow and residual bone graft were respectively 45.74 ± 0.09%, 48.09 ± 0.08%, and 6.16 ± 0.01%. The residual graft material appeared to be osteointegrated and none of the particles appeared to be encapsulated. The present case report supports the guidelines that assume that patients undergoing oral bisphosphonate therapy can be eligible for surgical therapy. More clinical studies with larger sample sizes are needed to support this clinical evidence.

## 1. Introduction

Loss of a tooth is a very frequent event generally as a consequent to caries and periodontal disease [1,2]. To date, the use of dental implants has become a well-accepted rehabilitation modality for patients who have lost their teeth [3]. However, dental extraction is also a cause of remodeling and resorption of the surrounding bone over time. These events lead to bone atrophy of the jaws and the largest dimensional variations occur within one year after extraction [2]. Clinical evaluations up to 12 months have demonstrated a loss of 3.87 mm in the crest width and 1.67 to 2.03 mm in crest height [4]. To overcome these dimensional changes, in recent years, several techniques and different types of biomaterials have been described and proposed. Alveolar socket preservation techniques have been demonstrated to be effective in reducing vertical and horizontal ridge alterations in post-extraction sites independently of the type of technique used [5]. In the literature, there are numerous studies that have evaluated bone regeneration following the use of different combinations of materials and surgical techniques [6,7,8,9,10,11]. It is well known that bone grafting reduces the resorption process occurring after tooth extraction [12], however, it is not yet clear which biomaterial is the most suitable for socket preservation; allografts, xenografts, and synthetic particulate materials have been extensively used and documented [13,14,15,16]. In this scenario, enzymatically-deantigenated equine bone has proven to be extremely biocompatible with new blood vessels in-growth during healing and is reported to be re-absorbed and replaced by newly formed bone a few months after insertion [17,18]. In addition, some studies have demonstrated that the combined use of biomaterials and membrane has achieved better results on post-extraction alveolar preservation [13]. In dental practice, clinicians often treat patients with comorbidities, including patients taking bisphosphonates (BPs). Oral BPs are most commonly used for the treatment of osteoporosis and osteopenia. They have been used in less common conditions, such as Paget’s disease of bone and osteogenesis imperfecta [19]. The BPs are incorporated into the bone matrix and the permanence in the tissue can last for several years [19]. In addition, BP treatment is strongly associated with a pathological condition called bisphosphonate-related osteonecrosis of the jaw (BRONJ), as reported in the position paper of the American Association of Oral and Maxillofacial Surgeons (AAOMS) [20]. According to a study conducted in Australia, in 2004 and 2005, the prevalence of BRONJ varied between 0.01% and 0.04% in osteoporotic patients [21]. In another study, a prevalence of 0.1% was reported in patients using oral BPs and this value increase to 0.21% in patients taking these drugs for over 4 years [22]. However, these data may not reflect the real situation, since the so called “stage 0” to differentiate patients who did not present the typical clinical signs found in osteonecrosis was only introduced in 2009 [19]. The incidence of osteonecrosis appears to increase with increasing duration of therapy with these drugs with considerable differences in relation to the type of drug, its potency, the route of administration (it is associated with a greater incidence when administered intravenously as compared with oral administration), and the underlying disease [22,23,24,25,26]. Among the surgical treatments, tooth extraction and socket preservation for implant-prosthetic rehabilitations are often required for patients affected by osteoporosis [19]. A recent review on osteoporotic patients taking oral BPs indicated that the incidence of osteonecrosis was low and was more common among female patients over 60 years old with previous invasive dental treatment [27]. Recent literature and general guidelines assume that dental surgery for patients under oral BPs is not completely contraindicated, but uncertainties and clinical issues still remain to be addressed [19], considering that the topic is poorly described in the literature. Therefore, the present case report of a patient under BP therapy aims at showing the histological results of the healing of a preserved extraction socket in humans.

## 2. Case Report

**Figure 1:**
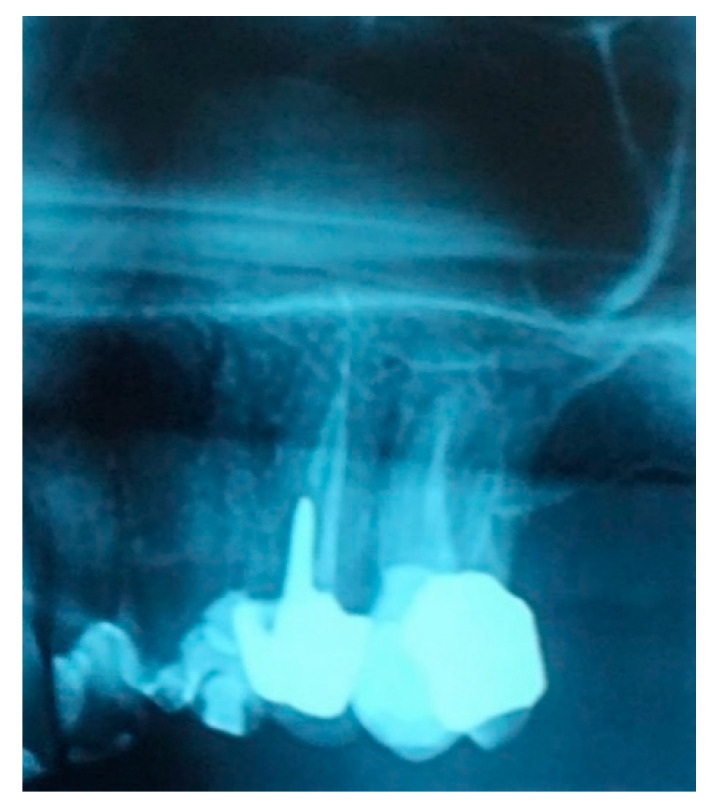
Detail of the patient’s orthopantomography (OPT) showing the first left upper molar candidate for extraction.

A 52-year-old osteoporotic patient came to our observation. The patient reported the first left maxillary molar painful during chewing. The clinical examination showed tooth and crown mobility and secondary decay under the crown. Radiographic examination showed an osteolytic lesion below the distal root due to an incongruous root canal treatment (Figure 1).

The best treatment we could provide to the patient was a tooth extraction and dental implant insertion to replace the tooth. In order to provide the following implant-prosthetic rehabilitation, post-extraction socket preservation was proposed. The patient referred that she had been under nitrogen oral bisphosphonates (BPs) (ibandronic acid, 150 mg) for osteoporosis since 2011. The patient referred to no history of cancer or radiotherapy, and no co-medications with steroids or anti-angiogenic drugs. The patient also reported having quit smoking. No pre-existing bone lesions or BRONJ were detected with preoperative orthopantomography (OPT). No bone exposures were observed during the clinical examination, and the patient did not show any symptoms of BRONJ. We were also informed that 3 years ago the patient underwent dental extractions and implant rehabilitation. The proposed plan of intervention included extraction of the hopeless tooth (26), socket preservation with enzymatically-deantigenated horse bone and horse pericardium membrane, and insertion of dental implants 5 months after extraction. The patient was informed about BRONJ potential risk, accepted the treatment plan and signed an informed consent. The BPs therapy was suspended before the start of the treatment. Precisely, the suspension was scheduled three months before the planned tooth extraction and resumed six months after prosthesis. Moreover, in preparation for the tooth extraction, the patient underwent an antibiotic treatment with amoxicillin 1 gr/twice a day for two weeks and used chlorhexidine 0.2% mouthwash during the week before the planned extraction. The tooth was extracted, and socket preservation was performed. The subsequent follow-up included clinical recalls every 30 days.

**Figure 2:**
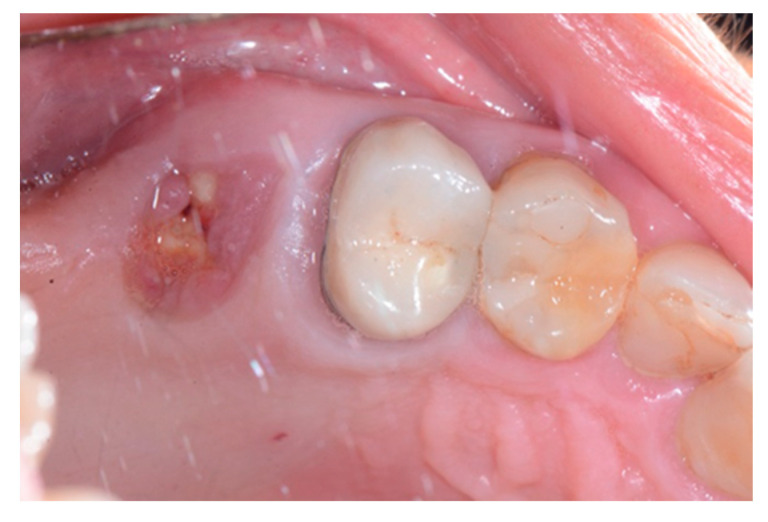
Clinical situation after crown removal.

**Figure 3:**
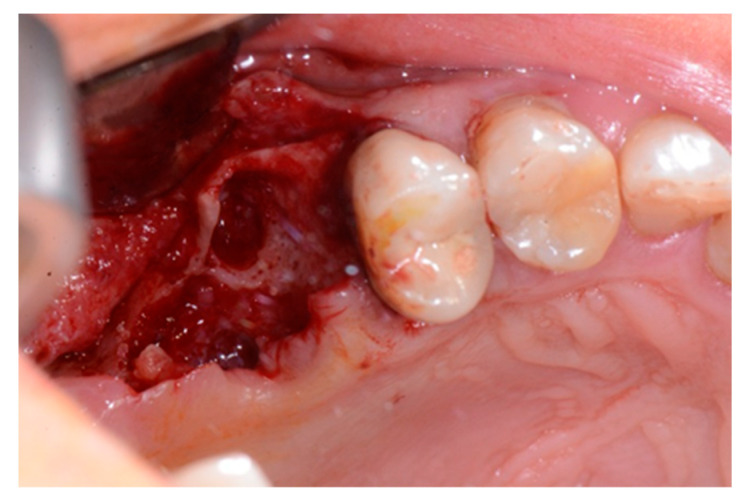
Alveolar socket after the extraction of the roots.

**Figure 4:**
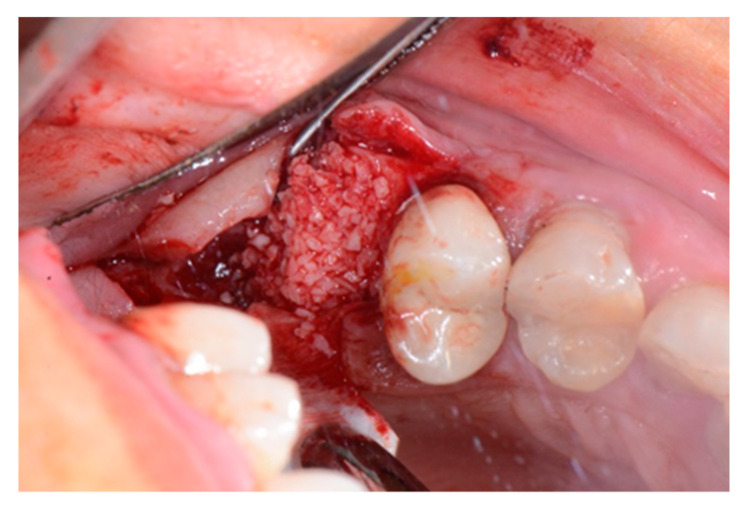
Alveolar socket filled with the graft material.

**Figure 5:**
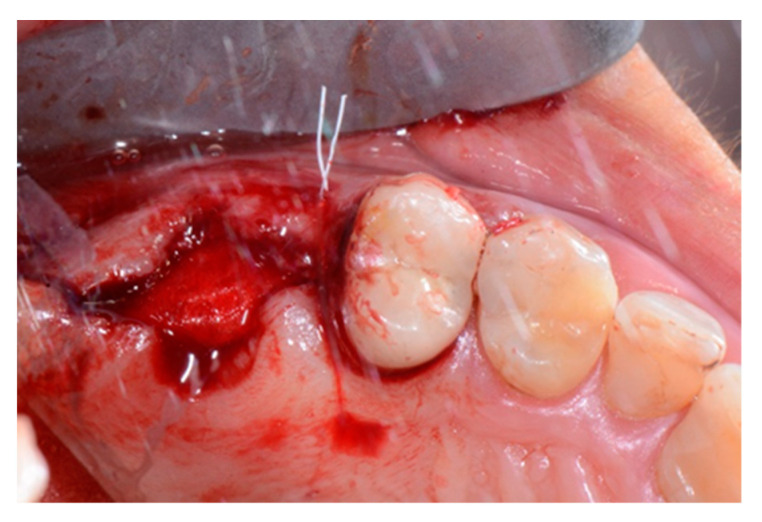
Intraoperative view of the alveolus after socket preservation. The preserved site is left to heal by second intention with the exposed membrane.

We used only surgical discarded material for the present study and the subject gave her informed consent before her participation. Seven days before the tooth extraction, the patient started prophylactic antibiotic therapy with amoxicillin/clavulanic acid (Augmentin, GlaxoSmithKline, Verona, Italy) and continued it for seven days after surgery 1 g/every 12 h, in addition to mouthwashes (chlorhexidine 0.2%) starting a week before surgery and continuing until healing occurred. Figure 2 shows the clinical situation after the crown removal. Once the extraction of tooth 26 (Figure 3) was carried out, the preservation of the alveolus was performed using a commercial enzymatically-deantigenated equine bone (Osteoxenon bone granules, Bioteck S.p.A, Vicenza, Italy) (Figure 4) and a commercial double layer of equine pericardium membrane (Heart pericardium membrane, Bioteck S.p.A., Vicenza, Italy) was used to cover and protect the site (Figure 5).

The patient was recalled every 30 days to evaluate the progression of the healing process and to avoid any early signs of complications. Five months after the extraction, cone-beam computed tomography was performed for insertion of the implant under local anesthesia and after antibiotic prophylactic treatment, as previously described. During the implant sites preparation, a bone biopsy was harvested using a 3 mm diameter trephine bur. After removal, the sample was immediately fixed in 10% buffered formalin, and then decalcified using Biodec-R solution (Bio-Optica, Milan, Italy), according to the manufacturer’s instructions. After these treatments, the sample was dehydrated with a series of alcohol rinses and embedded in paraffin for light microscope observations. The sample was cut longitudinally along the major axis using a microtome (Leica Microsystems Srl, Milan, Italy). Three slides of 6 µm were stained with Hematoxylin and Eosin and with Masson’s trichrome staining solutions.

Histomorphometric evaluations were carried out using a Zeiss Axioscope light microscope (Carl Zeiss AG, Oberkochen, Germany) equipped with a CoolSNAP videocamera (Photometrics, Tucson, AZ, USA) [28]. The optical system was associated with a MetaMorph Image Analysis system (Universal Imaging Corp, Downingtown, PA, USA), dedicated software with image-capture and analysis capabilities. The area occupied by bone tissue, marrow spaces, and residual graft material was evaluated measuring three slices from the sample. The morphometric analysis was carried out taking into consideration the ratio between bone surface and the whole area (bone/tot), the ratio between the marrow spaces and the whole area (marrow/tot), and the ratio between the residual graft material and the whole area (graft/tot). The mean value for each slice was calculated and compared.

## 3. Results

**Figure 6:**
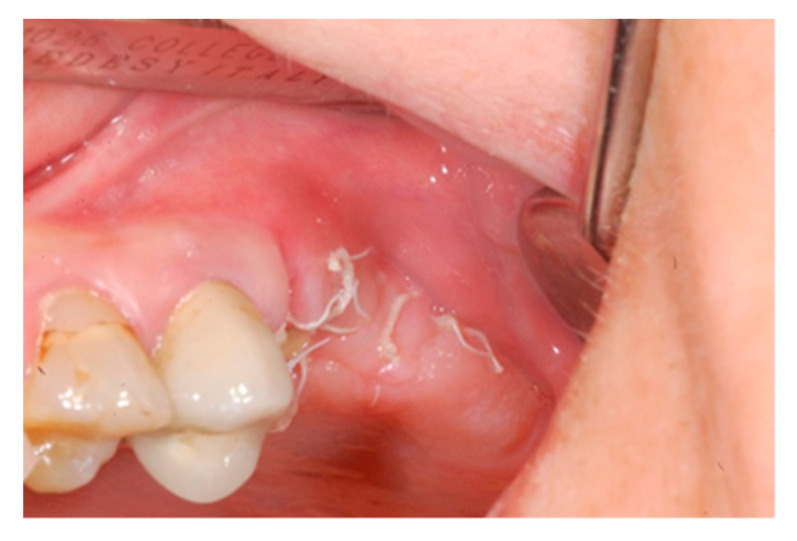
Vestibular intraoperative view of the oral mucosa 10 days after tooth extraction and socket preservation. Soft tissues appeared completely healed with no signs of bone exposition, considered as a clear sign of bisphosphonate-related osteonecrosis of the jaw (BRONJ).

**Figure 7:**
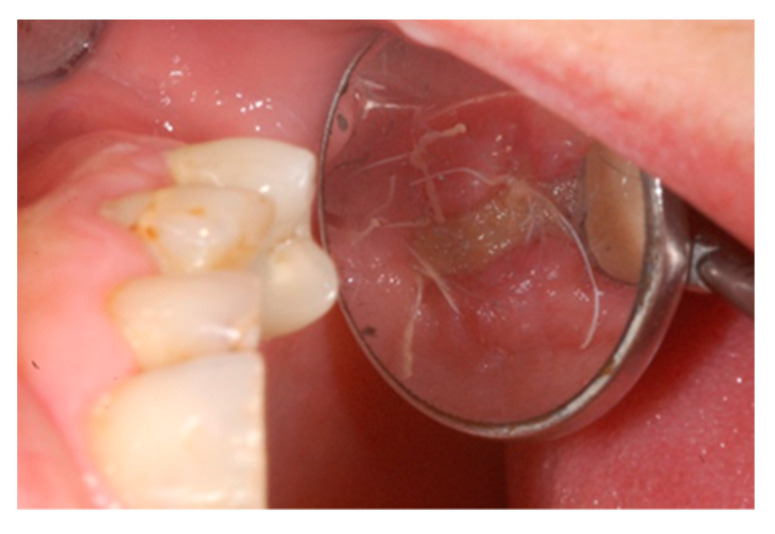
Occlusal intraoperative view of the oral mucosa 10 days after tooth extraction and socket preservation. The exposed membrane was covered by a fibrin layer. Sound margins of the wound are visible.

Ten days after the extraction and socket preservation, the patient was recalled for the suture removal. The clinical observation showed the occurrence of physiological healing without any complication (Figure 6 and Figure 7).

**Figure 8:**
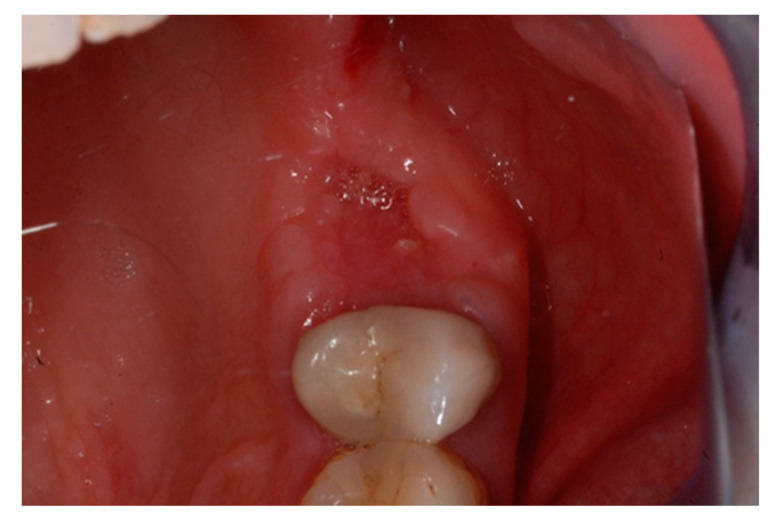
Intraoperative view of the oral mucosa at one month post tooth extraction. Soft tissues appeared to be completely healed with no signs of bone exposition, considered as a clear sign of BRONJ.

The one-month recall showed a full coverage of the site by epithelialized tissue, thus, indicating perfect healing (Figure 8).

**Figure 9:**
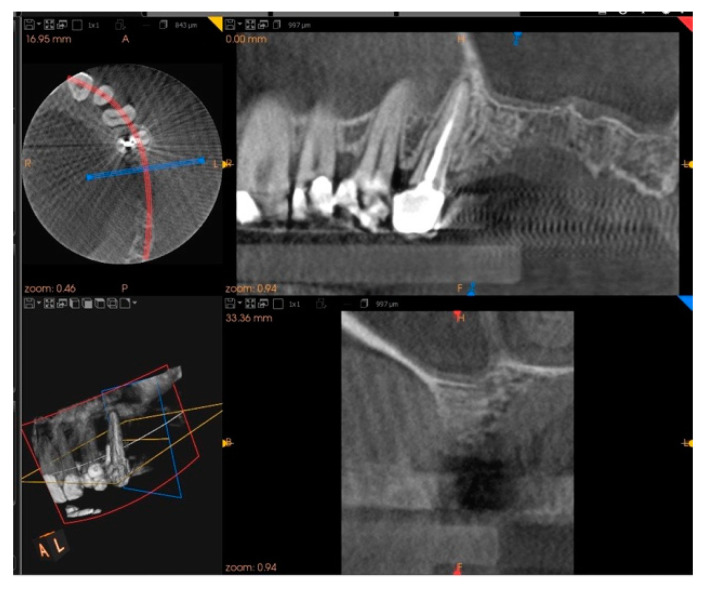
Cone-beam computed tomography (CBCT) shows bone volume preservation in the mesial surface of the alveolus.

**Figure 10:**
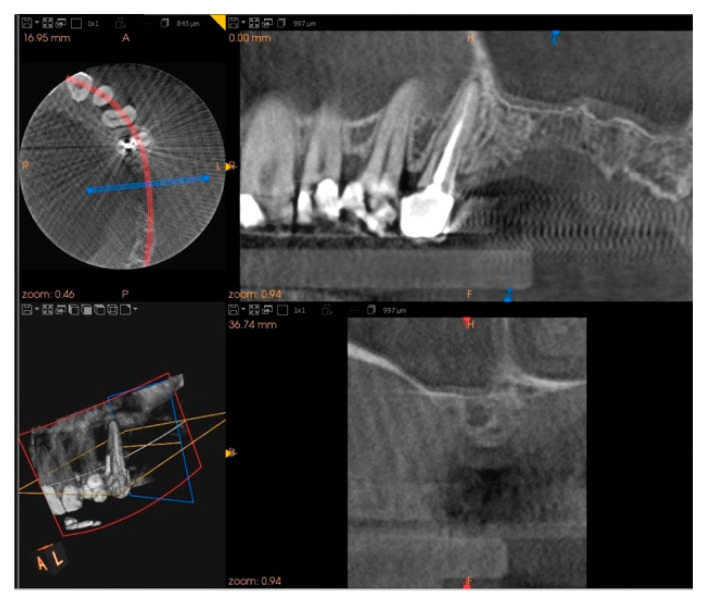
Cone-beam computed tomography (CBCT) shows bone volume preservation in the distal surface of the alveolus.

Five months after the extraction, a cone-beam computed tomography (CBCT) was also performed to design the implant insertion. The radiological examination showed bone volume preservation of the post-extraction site and no signs of osteonecrosis (Figure 9 and Figure 10).

### Histology

**Figure 11:**
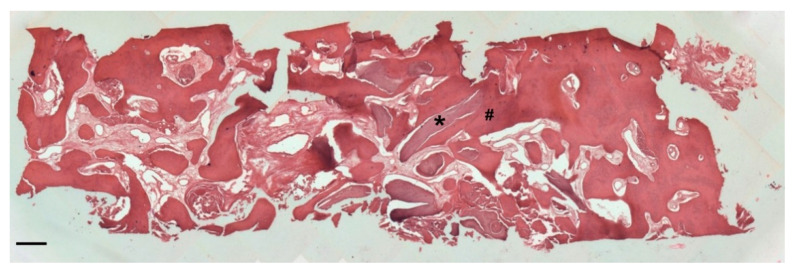
Two-dimensional (2D) reconstruction of the bone sample after staining with Hematoxylin and Eosin staining solution (magnification 25×, scale bar 400 μm). Bone trabeculae are dark-pink colored (#), whereas graft material appears light-pink colored (*).

At low magnification, trabecular bone appeared to be surrounded by loose bone marrow. The large component of newly formed bone surrounded the few granules of biomaterial not yet reabsorbed (Figure 11).

**Figure 12:**
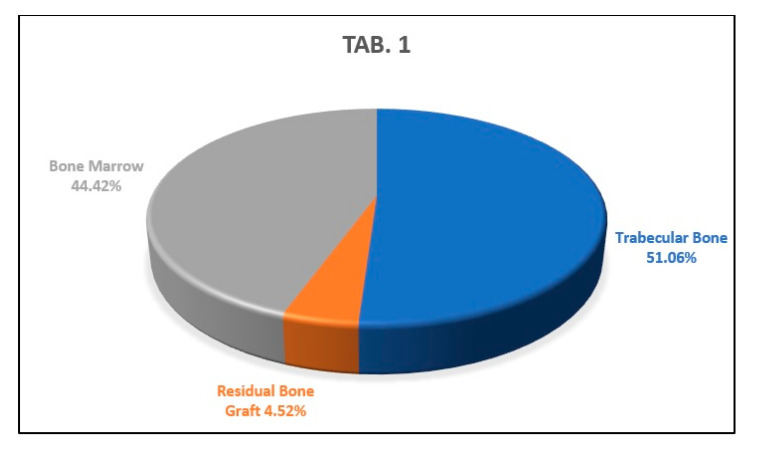
This graph shows the percentages of newly formed bone, bone marrow, and residual biomaterial granules in the coronal part of the analyzed sample.

Three serial sections of the sample were analyzed, taken in the coronal part, in the middle part, and in the apical part of the sample. From the analysis of the first histological section, we found that the percentages of newly formed bone, residual biomaterial, and bone marrow were, respectively, 51.06%, 4.52% and 44.42% (Figure 12).

**Figure 13:**
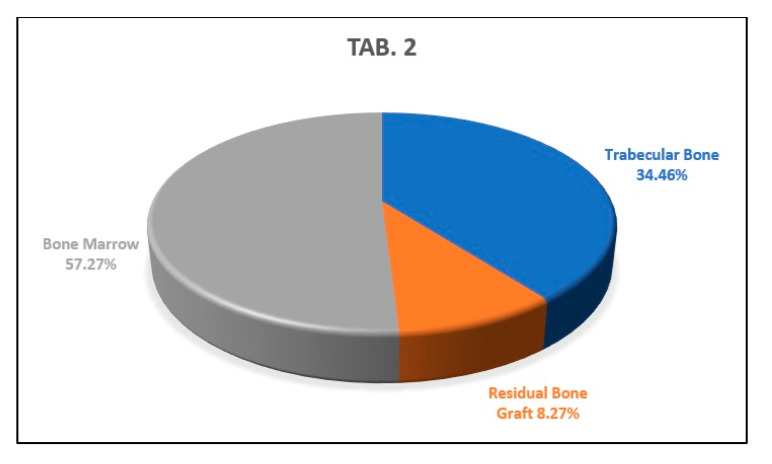
This graph shows the percentages of newly formed bone, bone marrow, and residual biomaterial granules in the medial part of the analyzed sample.

The analysis of the second histological section showed percentages of newly formed trabecular bone, residual biomaterial, and bone marrow of 34.46%, 8.27% and 57.27%, respectively, (Figure 13).

**Figure 14:**
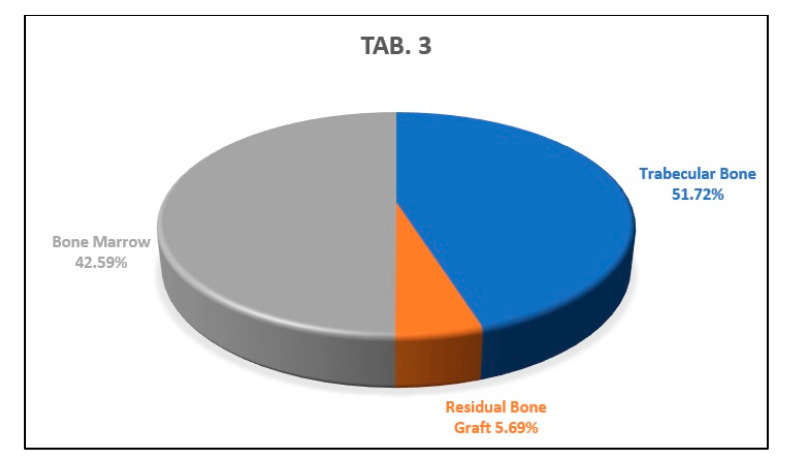
This graph shows the percentages of newly formed bone, bone marrow, and residual biomaterial granules in the apical part of the analyzed sample.

The analysis of the apical histological section showed a percentage of newly formed trabecular bone of 51.72% of the sample examined, a percentage of residual biomaterial of 5.69%, and a percentage of bone marrow area corresponding to the 42.59% of the histological sample (Figure 14).

**Figure 15:**
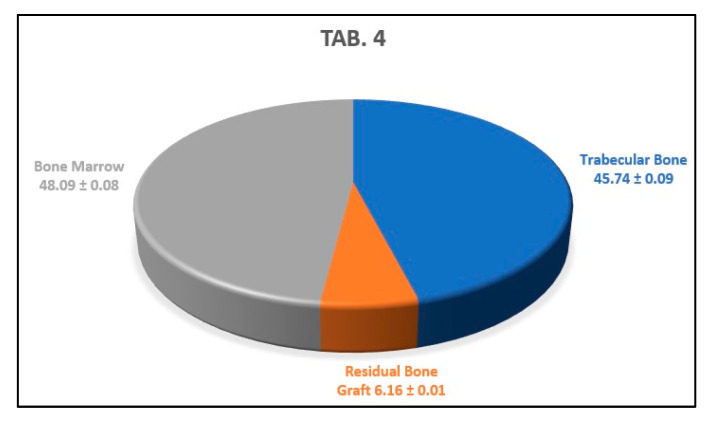
This graph shows the average percentages of newly formed bone, bone marrow, and biomaterial granules of the analyzed sample.

The average of the results obtained from the analysis of the previous three histological sections indicates that the newly formed trabecular bone area percentage was 45.74 ± 0.09%, the residual amount of biomaterial was 6.16 ± 0.01%, and the bone marrow area percentage was 48.09 ± 0.08% (Figure 15).

**Figure 16:**
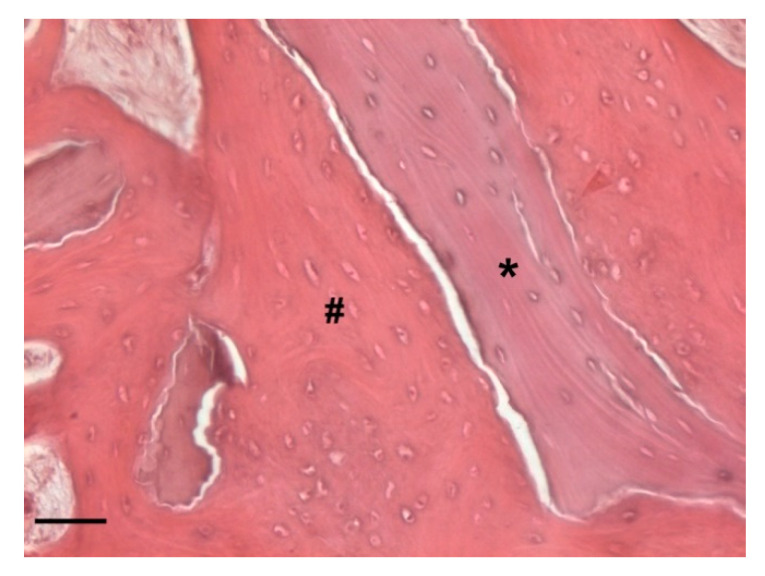
Detail of the bone sample stained with Hematoxylin and Eosin staining solution (magnification 100×, scale bar 100 μm). Graft material appears light-pink colored and with empty lacunae (*). The residual graft material appears detached from the bone surface due to a cutting artifact. Indeed, the bone graft particles appear to be completely osteointegrated and surrounded by woven bone (#). Big lacunae containing immature osteocytes are visible.

The periosteum showed no signs of hypertrophy. The bone resulted vital and no evidence of empty lacunae or morphological alterations to osteocytes was noted. The bone marrow space was occupied by loose connective tissue, rich in adipocytes and without signs of inflammatory infiltration and/or bacterial infection. The blood supply was guaranteed by numerous vessels close to the new trabecular bone and by haversian canals. The bone tissue from all the specimens showed features of mature lamellar bone, which was almost completely mineralized, although the presence of woven bone was also found. A lining layer of cells surrounded the whole bone surface. As shown in Figure 16, it was also clear that the area surrounding the graft material was embedded in trabecular bone and/or bone marrow connective tissue. The residual graft material appeared to be osteointegrated and none of the particles appeared encapsulated.

**Figure 17:**
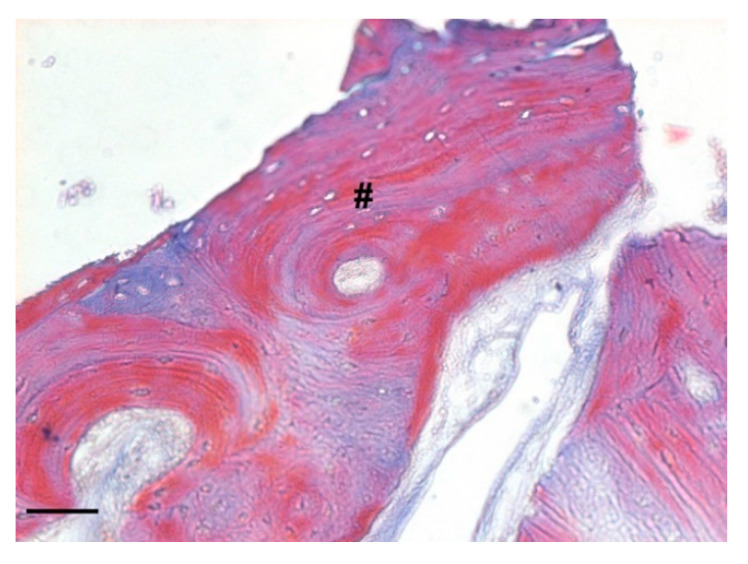
Detail of the bone sample stained with Masson’s trichrome staining solution (magnification 100×, scale bar 100 μm). The image shows the presence of lamellar bone indicating the complete bone remodeling (#).

Probable rows of osteoblasts in the active phase secreting osteoid tissue could be found in close proximity to the biomaterial granules. Moreover, in the deepest areas of the sample, the presence of lamellar bone, an index of complete bone remodeling, was found (Figure 17).

## 4. Discussion

Tooth extraction is the most common dental practice procedure consequent to caries or periodontal diseases [29] and the alveolar bone resorption is one of the main biological consequences connected to dental extraction. In the last years, several treatment modalities have been described to reduce the alveolar bone resorption [15] including immediate implants [30,31], the socket shield technique [31,32,33,34], the “all-on-four” treatment [35], and the alveolar ridge preservation technique [36,37]. In this scenario, pharmacological treatments may influence the successful outcome of implantation procedures connected to drug-related risks.

Bisphosphonates are a class of drugs used to treat many diseases such as osteoporosis, Paget’s disease, and bone metastasis. The literature has widely demonstrated that patients using BPs have an increased risk of developing complications due to dental treatments, such as BRONJ [19]. Nevertheless, a six-year clinical study that collected patients suffering from BRONJ showed that patients with osteoporosis taking oral BPs have much lower risk of developing osteonecrosis [38]. Oral surgical treatment is not contraindicated in this category of patients, but in our experience, each case must always be evaluated individually, and a detailed medical history of the patient is necessary to exclude possible factors that could expose the patient to a high risk of developing bone osteonecrosis. Before performing a surgical treatment, the patient must be informed of the risk of BRONJ and sign the informed consent before the start of the treatment. It is recommended to stop the BPs therapy three months before the oral intervention and to undergo a long antimicrobial prophylaxis treatment with antibiotics and chlorexidine mouthwashes [19,30]. After the treatment, a maintenance program with regular check-ups must be scheduled [39]. In recent years, considerable progress has been made regarding this topic, but there are still not many papers in the literature that provide histological data supporting clinical outcomes in humans. Our work has provided clinical and histological data regarding socket preservation in a patient with osteoporosis who was taking oral BPs. This treatment has a very important role in oral rehabilitation as it allows patients to maintain an adequate bone volume for an implant rehabilitation as already demonstrated in the animal model [40]. In our clinical case, a complete healing of the post-extraction socket treated with socket preservation technique was achieved in 5 months in clinical, radiological, and histological terms. Clinically, no bone exposures or BRONJ were detected during the follow-up period but, most importantly, histological analysis indicated a physiological healing of the preserved site. The average percentage of biomaterial present in the analyzed samples was very low as compared with the average percentage of the newly formed bone, indicating that 5 months after socket preservation, almost all the biomaterial had undergone remodeling, and was substituted by new trabecular bone. This data is in line with studies performed in animal model where 12 weeks after socket preservation procedures, vertical and horizontal changes of the residual alveolar ridge were diminished [41]. Our results demonstrate that the drug taken by the patient did not interfere with the cellular healing phases of the site treated with the socket preservation. Furthermore, the presence of the lamellar bone and the absence of grafted material in the deeper areas of the histological sample suggest that the bone remodeling phases have already occurred. Interestingly, the presence of cells (probably osteoblasts in the active phase) was found near the newly formed bone during the mineralization and grafting process, highlighting BPs role in bone remodeling, which involves the inhibition of osteoclast activity and activation of osteoblast function [42]. The histological analysis of our sample confirmed that the healing obtained with the socket preservation technique by using enzymatically-deantigenated equine bone and equine pericardium membrane is comparable to the healing obtained in patients who do not use bisphosphonates [17,18].

## 5. Conclusions

The present case report aligns with current literature, which assumes that patients receiving oral bisphosphonates therapy can be eligible for surgical dental therapy. The present case report showed no signs or symptoms of osteonecrosis during the ongoing follow-up. These conclusions are supported by the histological analysis which confirmed the correct healing of the alveolus and the presence of newly formed bone in close contact with the residual biomaterial granules. The data are encouraging, however, our results are based on only one clinical case, therefore, more studies are needed to strongly support our findings.

## Data Availability

The raw/processed data required to reproduce these findings cannot be shared at this time as the data also forms part of an ongoing study.

## Abbreviations

BPs	Bisphosphonates
BRONJ	Bisphosphonate-related osteonecrosis of the jaw
ONJ	Osteonecrosis of the jaw
AAOMS	American Association of Oral and Maxillofacial Surgeons
CBCT	Cone-beam computer tomography
OPT	Orthopantomography
